# Rubrene crystal field-effect mobility modulation via conducting channel wrinkling

**DOI:** 10.1038/ncomms7948

**Published:** 2015-05-05

**Authors:** Marcos A. Reyes-Martinez, Alfred J. Crosby, Alejandro L. Briseno

**Affiliations:** 1 Polymer Science and Engineering, University of Massachusetts Amherst, 120 Governor's Drive, Amherst, Massachusetts 01003, USA

## Abstract

With the impending surge of flexible organic electronic technologies, it has become essential to understand how mechanical deformation affects the electrical performance of organic thin-film devices. Organic single crystals are ideal for the systematic study of strain effects on electrical properties without being concerned about grain boundaries and other defects. Here we investigate how the deformation affects the field-effect mobility of single crystals of the benchmark semiconductor rubrene. The wrinkling instability is used to apply local strains of different magnitudes along the conducting channel in field-effect transistors. We discover that the mobility changes as dictated by the net strain at the dielectric/semiconductor interface. We propose a model based on the plate bending theory to quantify the net strain in wrinkled transistors and predict the change in mobility. These contributions represent a significant step forward in structure–function relationships in organic semiconductors, critical for the development of the next generation of flexible electronic devices.

## Abstract (web summary)

Building flexible electronic devices demands an understanding of how mechanical deformation affects the active materials. Here, the authors observe and quantify the effects of local strains generated by the wrinkling instability in the conducting channel of rubrene crystal thin-film transistors.

## Graphical abstract

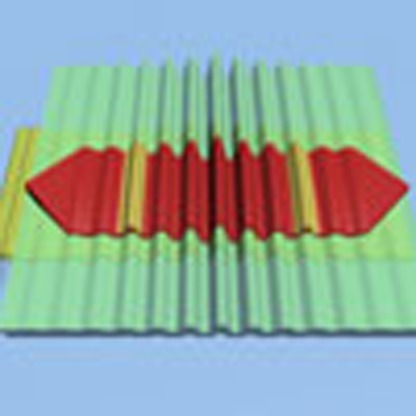

The organic electronics community has achieved unprecedented progress understanding the fundamental mechanisms of charge transport in organic materials through the study of molecular crystals^1^,^2^,^3^,^4^. Given their well-ordered structure, their purity and the absence of grain boundaries, organic single crystals (OSCs) allow almost complete experimental access to the intrinsic properties of the material in question. Significant efforts into studying the effects of global bending on the field-effect mobility of organic polycrystalline films can be found in the literature. Sekitani *et al*.^5^,^6^ studied the effects of bending on the performance of pentacene field-effect transistors fabricated on flexible PEN substrates. They showed that bending strains can affect field-effect mobility. Recently, Podzorov and coworkers^7^ demonstrated the mechanical robustness of thin, flexible OFETS of bis-(triisopropylsilylethynyl) pentacene and bis-triethylsilylethynyl anthra-dithiophene by bending them multiple times without degradation of performance.

Few publications on the effects of strain on the electrical properties of OSCs have been reported. Bao and coworkers^8^ fabricated rubrene field-effect transistors on plastic substrates, applied global bending of different bending radii and observed how the device performance changed. Degradation, associated to cracks in dielectric layer, was observed above certain tensile strains. Rang *et al*.^9^,^10^ studied the hydrostatic pressure dependence of charge carrier transport by measuring changes in the photoconductivity of tetracene, pentacene and rubrene single crystals. An increase in photoconductivity was observed and ascribed to the reduction of intermolecular distances due to pressure. More recently, Takeya and coworkers^11^,^12^ have further studied the deformation of OSCs under hydrostatic pressure and the effects on the electronic properties of crystals. To date, there has not been any research investigating the performance of single-crystal field-effect transistors (SCFETs) while undergoing inhomogeneous bending strains focused in the conduction region, which is located within the first few molecular layers above the dielectric/semiconductor interface^13^.

We take advantage of the wrinkling instability of thin films on soft substrates as a unique way to strain the conducting channel of field-effect transistors in a non-destructive, reversible and predictable manner. Wrinkling is a mechanical instability that occurs when a thin, stiff film adhered to a relatively thick, soft, elastic substrate is compressed in plane above a critical strain value^14^,^15^. Wrinkle formation is spontaneous, producing periodic, wavy topographic patterns. For the case of uniaxial strain, the wrinkling morphology follows a one-dimensional sinusoidal pattern, which applies both local compressive and tensile strains to the capping film in a continuous fashion. Recently, wrinkling was effectively used as a metrology tool to map the elastic constants of rubrene single crystals along different crystal directions^16^.

Here we demonstrate that the high field-effect mobility is maintained in rubrene single-crystal transistors undergoing inhomogeneous strain conditions. Changes in field-effect mobility, both increases and decreases, are observed on wrinkling. We discover that this change in performance is dictated by the cumulative effect of local deformations, net strain, at the dielectric–semiconductor interface. We propose an analytical model based on the plate bending theory to quantify the net strain in wrinkled conducting channels of transistors and predict the change in mobility. The present study is, to our knowledge, the first report demonstrating the wrinkling of high-performance organic single-crystal field-effect transistors and the impact of local bending of the conducting channel in charge transport characteristics.

## Results

### Field-effect transistor characteristics

**Figure 1:**
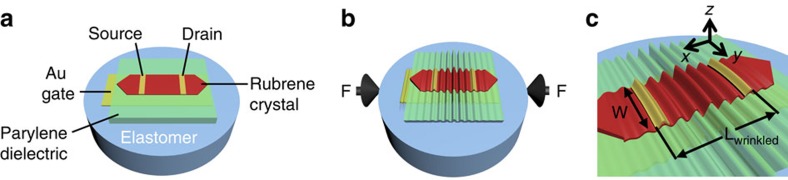
Rubrene single-crystal transistor on elastomeric substrate. (**a**) Structure of rubrene single-crystal transistor. The crystal is embedded in parylene dielectric only exposing the (100) rubrene crystal facet. (**b**) The transistor wrinkles at a critical global compressive strain. The present study utilizes uniaxial compression along the high-mobility axis [010] of rubrene crystals. (**c**) Coordinate system. The plane of the transistor corresponds to the *x*–*y* plane, which is perpendicular to the *z* axis.

Figure 1 shows the structure of the rubrene SCFET on an elastomeric substrate used in this study. Detailed fabrication steps can be found in Supplementary Fig. 1. Thin rubrene crystals (200 nm–1 μm), grown using the physical vapour transport method^16^,^17^, are utilized as the active layers for all transistors. The use of a cylindrical elastomeric substrate allows the precise alignment of the direction of applied strain by rotation. This study focused on the effects of wrinkling along the direction of high mobility in rubrene ([010]), also referred to as the *b* axis^4^,^18^. Parylene is used as the insulating layer due to the demonstrated low density of traps at the dielectric/crystal interface^2^,^19^,^20^,^21^. Parylene films are transparent, pinhole free and possess exceptional mechanical (Young's modulus=2.4 GPa, yield strength=42 MPa (Specialty Coating Systems Inc.)) and dielectric properties (*ɛ*=2.65, breakdown electric field of 10 MV cm^−1^ for 100 nm thick films)^22^. For this study, we utilize a top contact geometry to ensure intimate metal/semiconductor interface while applying mechanical strains. All of the transistors fabricated for this study exhibit excellent characteristics, with mobilities as high as 8 cm^2^(Vs)^−1^, *I*on/*I*off of 10^6^ and threshold voltages close to 0 V. Typical device output and transfer characteristics for planar and wrinkled devices are shown in Supplementary Fig. 2.

### Contact resistance effects

**Figure 2:**
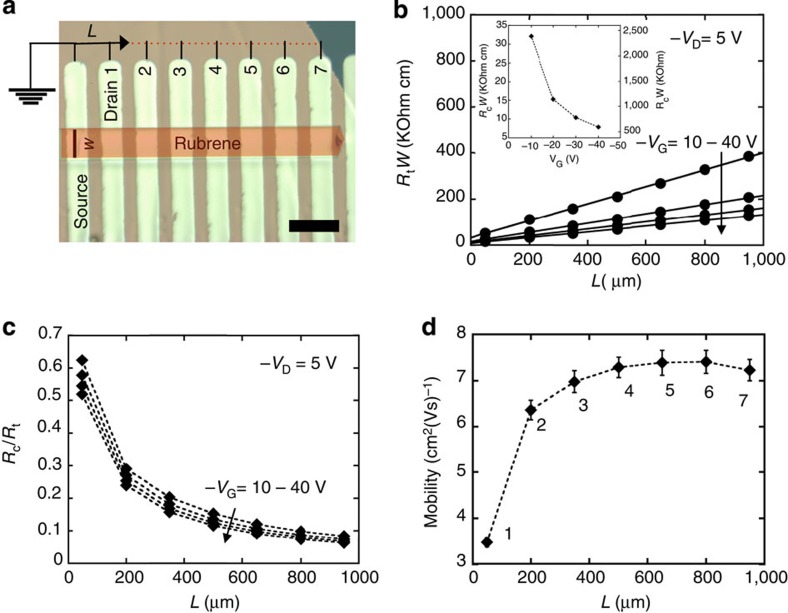
Contact resistance determination. (**a**) Optical micrograph of representative single-crystal FET with multiple top contact source and drain electrodes allowing for multiple channel length testing on same crystal sample. Length of the scale bar is 200 μm. The crystal is colourized for clarity. For additional details, see Supplementary Table 1. (**b**) Width-normalize total device resistances (*R*_t_) as a function of channel length for different gate voltages. The contact resistance (*R*_c_) as a function of gate voltage is shown in the inset. (**c**) Fraction of total device resistance due to contact as a function of channel length for different gate voltages. (**d**) Mobility trend as a function of channel length and channel aspect ratio (*L*/*W*) for the device in **a**. Mobility changes are negligible above a channel length of ∼500 μm. Error bars in mobility correspond to the s.e.m. from six independent measurements at different drain voltages in the linear regime, −5 to −15 V at −2 V steps.

Considerations are taken to ensure that the charge carrier mobilities measured are not contact resistance limited. It is known that channel length affects the total device resistance in two-probe measurements^23^,^24^. To determine how mobility is affected by channel length, multiple parallel gold contacts were deposited through a shadow mask on the single crystals, allowing for multiple field-effect mobility measurements on the same crystal sample as a function of channel length. Figure 2a shows a micrograph of a representative rubrene SCFET with multiple contacts. Devices were measured by using one contact as the common source electrode and the remaining contacts as drains, one at a time, systematically increasing the channel length. We observe that, for the same crystal, field-effect mobility increases with increasing channel length, with negligible change observed above length of ∼500 μm after which the changes are not as pronounced. This trend can be explained by considering the total resistance, *R*_t_=*R*_c_+*R*_ch_, as a function of the contact resistance *R*_c_ and the channel resistance *R*_ch_. *R*_ch_ changes with channel dimensions according to *R*_ch_=*ρ*(*L*/*Wt*) (ref. 25), and gate voltage *V*_G_. Therefore, for the same *V*_G_, mobility changes with channel dimensions because the relative magnitude of *R*_ch_ increases and the influence of *R*_c_ is reduced as the channel length increases. The intrinsic channel mobility dominates transport at large channel lengths while contact resistance limits it at short lengths. The 500-μm channel length is not a general rule for non-contact-limited mobility; a more accurate indicator is the channel aspect ratio (*L*/*W*). All the mobilities measured plateau at an *L*/*W* value of ∼2.5 (see Supplementary Fig. 3). Contact resistances were determined using the gated transmission-line method^24^,^26^. The width-normalized total resistances (*R*_t_*W*) as a function of channel length is shown in Fig. 2b. Figure 2c shows the fraction of the total device resistance due to contact resistance (*R*_c_/*R*_t_). We observe that at channel lengths >500 μm, the contact resistances correspond to <20% of the total resistance measured. In Fig. 2d, the trend of mobility as a function of channel length and channel aspect ratio is shown for the same crystal sample shown in Fig. 2a. Given the contact-limited mobility trend observed in other rubrene SCFET tested in this study (see Supplementary Fig. 3), only devices with negligible dependence of mobility on channel length were utilized for wrinkling experiments.

### Field-effect mobility modulation

**Figure 3:**
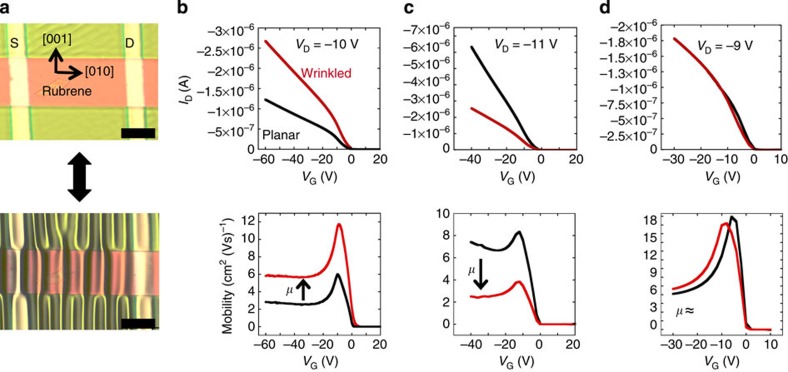
Field-effect mobility modulation via conducting channel wrinkling. (**a**) Optical micrograph of single-crystal FET in its planar and wrinkled configurations with top source (S) and drain (D) electrodes. Length of the scale bar is 100 μm. (**b**–**d**) Drain current *I*_D_ versus gate voltage *V*_G_ corresponding to three different devices in their planar configuration and while undergoing wrinkling. Corresponding mobility versus gate voltage is shown. For experimental details on individual devices tested, see Supplementary Table 1.

Applying in-plane, uniaxial, global compressive strains (
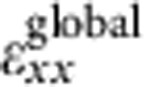
) above a critical value, generates one-dimensional wrinkling across the conducting channel of the SCFET. 
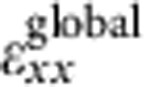
 is defined by the change in diameter (*D*_cyl_) of the PDMS substrate in the direction of compression as 
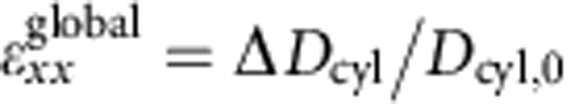
. Figure 3a shows the optical micrographs of a rubrene single-crystal device in its planar and wrinkled configurations. For small 
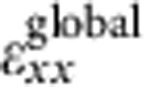
 (
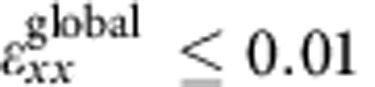
 for devices in this study), wrinkling is reversible such that when the compression is removed, wrinkles disappear. At large 
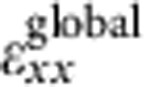
 values, delamination of SCFET from PDMS substrate occurs (Supplementary Fig. 4). Despite maintaining excellent transistor characteristics while undergoing wrinkling (Supplementary Fig. 2), impact in field-effect mobility is observed. Figure 3 shows comparative transfer characteristics between planar and wrinkled devices along with the corresponding plots of mobility, *μ*, as a function of gate voltage, *V*_G_. Significant increase (Fig. 3b) and decrease (Fig. 3c) in mobility can be measured when SCFETs are wrinkled. Other devices show almost negligible change on wrinkling (Fig. 3d). As a control, we determined that the capacitance of parylene is unchanged under the applied strain in our experiments (Supplementary Fig. 5); therefore, the same capacitance values are used to calculate the mobility of both planar and wrinkled devices.

It has been shown that that the coexistence of different d-spacings in an organic semiconductor can affect the mobility by inducing shallow trap states. Examples of this effect can be seen in pentacene OFETs^27^ and in organic thin-film transistors prepared using the vibration-assisted crystallization method introduced by Diemer *et al*.^28^. The strain in a wrinkled SCFET is inhomogeneous; therefore, an array of different interplanar distances must be present. To test the impact of this phenomenon, we estimated the trap density, *N*_it_, for the devices shown in Fig. 3 using the expression^29^: 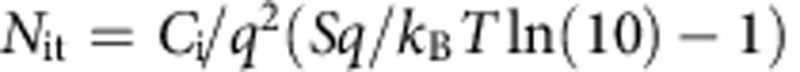, where *C*_i_ is the capacitance of the insulator, *q* is the elementary charge, *S*=d*V*_G_/d(log *I*_D_) is the subthreshold slope, *k*_B_ is the Boltzmann constant and *T* is temperature. Small differences in trap-state densities are observed between the planar and wrinkled configurations of all devices (Supplementary Note 1), including a reduction in the trap density for the case of mobility decrease shown in Fig. 3c. No correlation is apparent between the increase/decrease of the density of interfacial trap states and mobility, which indicates that this effect is not dominant in the change in mobility observed on wrinkling.

### Strain analysis in wrinkled conducting channel

**Figure 4:**
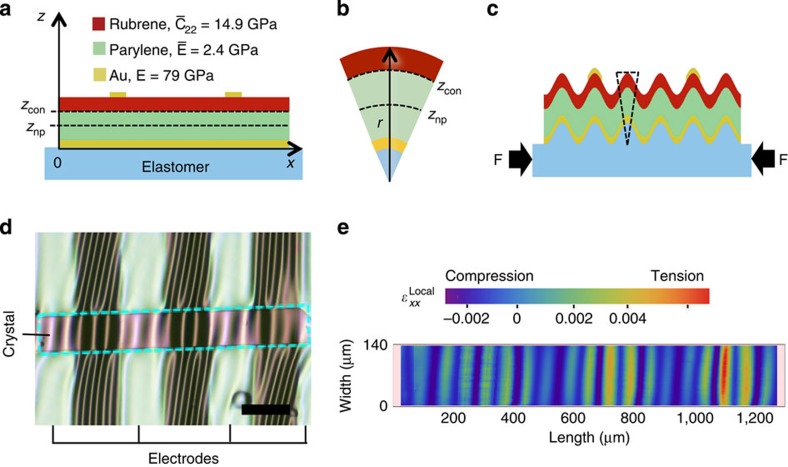
Net channel strain analysis. (**a**) Cross-section of single-crystal transistor structure. Charge transport in field-effect transistors occurs at the dielectric/semiconductor interface (conducting channel, *z*_con_). The position of neutral plane, *z*_np_, can be manipulated by changing the thicknesses of the different layers in the device. (**b**) The local channel strain, 
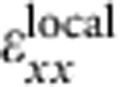
, will depend on the position of *z*_con_ with respect to *z*_np_ and local out-of-plane deflection. (**c**) Wrinkled transistors can be treated as a bending composite plate with continuously changing curvature. (**d**) Optical micrograph of wrinkled rubrene single-crystal transistor. Length of the scale bar is 200 μm. For additional details, see Supplementary Table 1. (**e**) Visualization of local strain, 
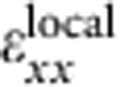
, corresponding to the channel area dotted area in **d**, including area under source and drain electrodes.

To understand the effects of wrinkling on mobility, we hypothesize that the different behaviours observed depend on the net strain in the [010] direction (*x* axis) experienced by the conducting channel while wrinkling. SCFETs are multilayered structures composed of different materials spanning a large range of thicknesses and mechanical properties. Figure 4a shows a cross-sectional schematic of the device indicating the positions of each layer, the position of the conducting channel, *z*_con_, and the representative position of the neutral plane, *z*_np_, which is the plane that remains unchanged in length on bending. Ignoring shear stresses at the PDMS/SCFET interface, we can consider the wrinkled SCFETs as composite plates that are undergoing bending with continuously changing curvature (Fig. 4b,c). Continuous changes in concavity in the wrinkled channel translate into continuous local change between mechanical tension and compression. The local strain of a wrinkled plate is written as a combination of the contributions of the von Kármán non-linear plate model^30^ and the local bending^31^,^32^:


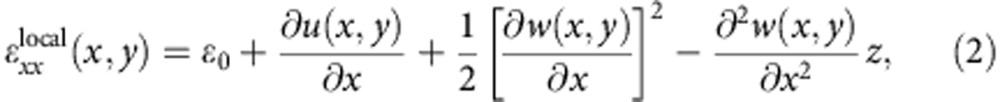


where *ɛ*_0_ is the critical strain for buckling, *u* is the in-plane displacement in the *x* direction, *w* is the out-of-plane deflection and *z* is the vertical coordinate through the thickness of the SCFET. In our experiments, the direction of wrinkling corresponds to the *x* axis (Fig. 1c). For a wrinkled plate with small out-of-plane deflections, the in-plane displacement gradient is negligible. In the present device structure, *z* represents the position of the conducting channel with respect to the neutral plane. Assuming that the charge carrier conduction is restricted to the dielectric/crystal interface in all devices, both in their planar and wrinkled configurations, makes *z* constant: *z*=*z*_con_−*z*_np_. For the pure bending of a freestanding, multilayered film, the neutral plane location is determined by:


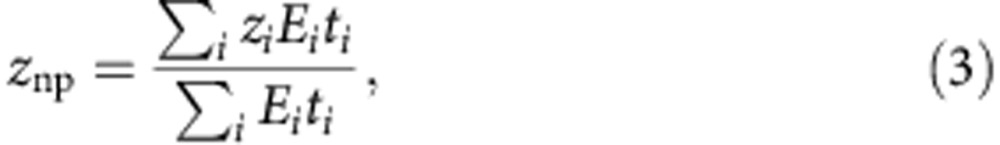


where *z*_*i*_ is the distance of centroid of the *i*th layer, *E*_*i*_ and *t*_*i*_ are the Young's modulus and thickness of the corresponding layer, respectively.

The topography of wrinkled transistors is measured using optical profilometry (see Supplementary Fig. 6). The profilometry data correspond to the out-of-plane deformation of the top surface, but for an accurate determination of the bending strain, a correction of the last term in equation (2), corresponding to the curvature of the neutral plane, is required. A detailed derivation of the corrected form can be found in Supplementary Note 2. The final form of 
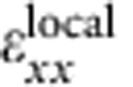
 is:


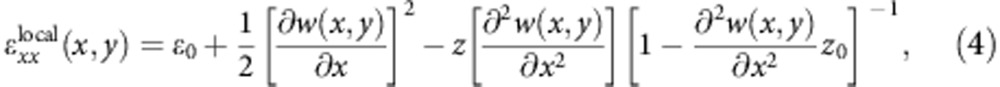


where *z*_0_ is the distance between the top surface of the transistor and the neutral plane. Equation (4) allows us to evaluate the local strain everywhere in a wrinkled conducting channel, provided the local out-of-plane displacement values and the critical strain for buckling, *ɛ*_c_=*ɛ*_0_ are known. *ɛ*_0_ was calculated from the device wrinkle wavelength at low strain using the classical wrinkling relation^33^: 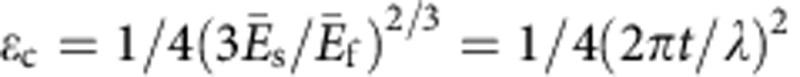. Figure 4e shows a density map of strain corresponding to the dotted area on the wrinkled transistor on Fig. 4d. It has been shown that the global bending of organic transistors based on polycrystalline thin films can have different effects in the field-effect mobility^5^,^6^,^34^; therefore, we infer that different local strains in the wrinkled channel have different effects in the field-effect mobility. The main difference is that the distribution of strains in a wrinkled transistor is inhomogeneous; hence, a way to quantify the net strain in a wrinkled channel is required. We propose superposing each local strain contribution across conducting channel normalized by the projected area of the wrinkled channel, *WL*_wrinkled_, to obtain a net strain value, 
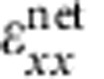
, in a wrinkled transistor:


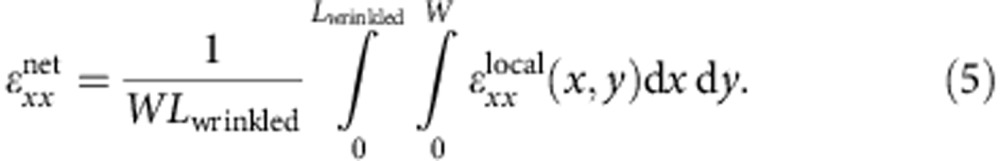


**Figure 5:**
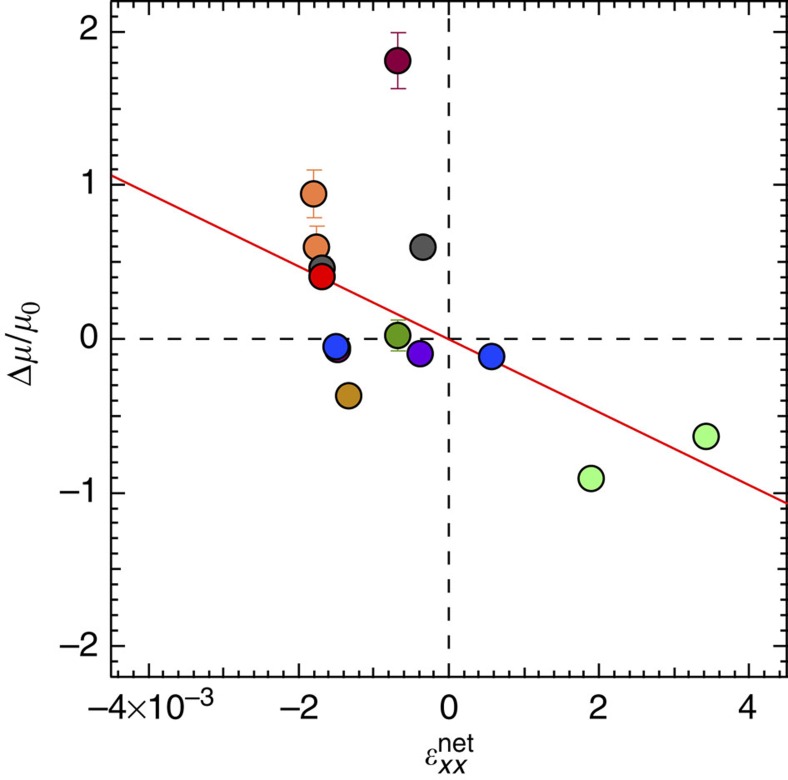
Effect of net channel strain in field-effect mobility. Net compression (negative values) causes mobility increase and the opposite effect is observed for net channel tension (positive values). Different colour markers represent different devices. The s.e. in 
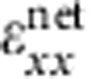
, calculated from the uncertainty of *ɛ*_0_ and 
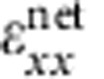
 integration limits, is not visible in the present scale. Error bars in mobility change correspond to the s.e.m. from six independent measurements at different drain voltages in the linear regime, −5 to −15 V at −2 V steps. For experimental details on individual devices tested, see Supplementary Table 1.

The 
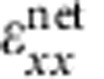
 value can be used to understand the effect of complex mechanical deformations on field-effect mobility. Figure 5 summarizes the mobility change as a function of 
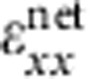
 for different crystal samples. We discovered that, for negative 
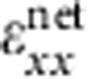
 values, mobility increases because the wrinkled channel is in a state of net compression. Positive 
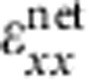
 values cause the mobility to decrease due to a state of net tension.

## Discussion

Our analysis assumes that the effect of strain on mobility follows the same linear trend for both tension and compression. The rubrene molecules are bonded by weak van der Waals forces in the solid state. Therefore, in comparison with its covalently bonded inorganic counterparts, rubrene single crystals can be considered soft semiconductors. Previous efforts by Sekitani *et al*.^9^,^10^ studying the effects of bending strains on OFET mobility and Rang *et al*.^10^ studying the effect of hydrostatic pressure, attribute changes in electrical performance to the change of intermolecular spacing. We speculate that bending strains in wrinkled transistors will affect the intermolecular distances in the crystal and consequently the electrical properties of the SCFETs. Mechanical compression will reduce the intermolecular distances, increasing mobility and tension will increase them, decreasing mobility. It is important to mention that our model does not consider the effects of strain on charge injection. If by straining, we are indeed modifying the d-spacing along the conducting channel, we expect charge injection to be affected. In order for this phenomenon to be integrated into our model, we would need to understand how changing crystal structure affects charge injection and the current density through the thickness of the crystal along the path from the electrode/organic semiconductor interface to the organic semiconductor/insulator interface. In our analysis, we include the area under source and drain electrodes, as we hypothesize that the current density under the contacts is affected by mechanical deformation.

In conclusion, the wrinkling of rubrene field-effect transistors demonstrates the ability of OSCs to maintain high performance while withstanding inhomogeneous strains in the conducting channel. Changes in mobility due to wrinkling reveal that the performance of rubrene is very sensitive to small deformations of the conducting channel. We propose a model based on plate bending to quantify the net strain in the dielectric/crystal interface of wrinkled rubrene transistors. One important finding of this work is the ability to relate the change in mobility with both compressive and tensile net strains in the conducting channel. Although further work is required at the intersection of mechanics of materials and organic electronics to define the limits of performance of organic semiconductors under mechanical strain, these results provide a strong foundation for the potential of using the wrinkling instability to modulate, even enhance, the performance of organic semiconductors which benefit from uniquely soft intermolecular bonds. This work will have broad applicability, bringing new understanding of strain-induced changes in performance observed when deforming different device structures.

## Methods

### Crystal growth

The process of horizontal physical vapour transport was used to grow rubrene single crystals. Commercially available rubrene (Acros Organic) was used as the source material for crystal growth. The as-received rubrene was purified three times through physical vapour transport before utilizing as an active material in transistors. The third crystallization step followed rapid crystal growth conditions to obtain very thin, flat crystal platelets. These conditions were achieved by a fast sublimation rate of the rubrene powder at a temperature of 330 °C under a flow of Argon at a rate of about 100 ml min^−1^. The average time for growth under these conditions is ∼30 min starting from room temperature.

### Transistor fabrication

First, the single crystal is manually laminated on a poly(acrylic acid) (PAA)-coated silicon wafer. PAA is a water-soluble polymer that functions as a sacrificial layer for lift-off. Next, parylene-N film (Specialty Coating Systems) is deposited on the crystal. Parylene serves as gate-dielectric material in the transistor structure. A 40-nm gold layer is subsequently thermally evaporated on the surface of the parylene film to act as the gate electrode. The Au/parylene/crystal configuration is then stamped on the surface of a cylindrical PDMS (Dow Corning Sylgard 184) substrate using a small flake of graphite to allow electrical contact with the bottom gate. The PAA is easily dissolved by partially submerging the complete device assembly in water. Once the PAA is dissolved, gold source and drain electrodes are thermally deposited on the now-exposed (100) facet of the rubrene crystal. The transistors are fabricated in such a way that the active layer is completely embedded in the dielectric and only one facet of the crystal is exposed. Notice also that the fabrication process places all electrodes and dielectric directly on the surface of the crystal. This minimizes interfacial defects that come from lamination and coating of the different layer on prefabricated substrates.

### Wrinkling by mechanical compression

Two Thorlabs one-dimensional translation stages were utilized to build a strain stage. The PDMS cylinder with the crystal attached to its top is placed between the two translating blocks for compression.

### Transistor characterization

To ensure a homogeneous charge carrier density across the conducting channel all FETs were operated in the linear regime^35^ with drain voltages *V*_D_ ranging from −5 to −15 V, following the expression: 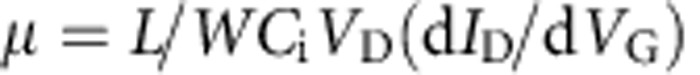, where *C*_i_ is the capacitance per unit area of the dielectric, *V*_G_ is the gate voltage, *V*_T_ is the threshold voltage and *μ* is the field-effect mobility. Mobilities were extracted from the non-*V*_G_-dependent mobility region of the *μ* versus *V*_G_ plot.

### Equipment and settings

Crystal thickness and topographic data were obtained by optical profilometry (Zygo NewView 7300). Parylene thickness was measured by spectral reflectance (Filmetrics F20). Optical micrographs were obtained with a Zeiss Axio Scope A1 equipped with Zeiss AxioCam ICc1 camera.

## Author contributions

M.A.R.-M. conceived and carried out the experiments, developed the fabrication process for SCFETs, produced the samples and analysed the data. A.J.C. and A.L.B. informed and directed the research. M.A.R.-M. wrote the manuscript in collaboration with all the authors.

## Additional information

**How to cite this article:** Reyes-Martinez, M. A. *et al*. Rubrene crystal field-effect mobility modulation via conducting channel wrinkling. *Nat. Commun.* 6:6948 doi: 10.1038/ncomms7948 (2015).

## Supplementary Material

Supplementary Information Supplementary Figures 1-8, Supplementary Table 1 and Supplementary Notes 1-2
