# Supplementary material for: Rubrene crystal field-effect mobility modulation via conducting channel wrinkling
Source: Nat Commun. 2015 May 5;6:6948. doi: 10.1038/ncomms7948 (PMC4432628; doi:10.1038/ncomms7948)
Supplement: Supplementary Information — Supplementary Figures 1-8, Supplementary Table 1 and Supplementary Notes 1-2 [file ncomms7948-s1.pdf]

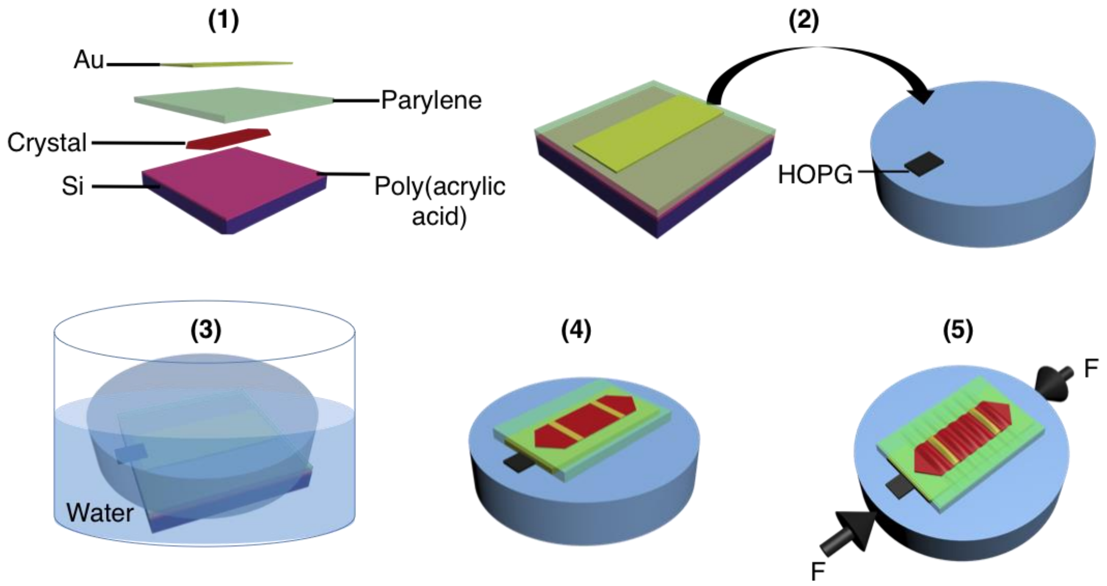

Supplementary Figure 1. Fabrication of single-crystal transistor on elastomeric substrate. (1) Rubrene crystal is laminated on Poly(acrylic acid) (PAA) coated Si wafer. Parylene is vapor- deposited on crystal and then gold gate is thermally evaporated. (2) The complete FET assembly is laminated on elastomer substrate with gate electrode facing the bottom. An HOPG flake is used to facilitate contact with the gate electrode. (3) The elastomer/FET is partially submerged in water to dissolve PAA layer and expose the top facet of crystal. (4) Top source and drain Au contacts are evaporated using a shadow mask. (5) The completed FET is able to wrinkle.

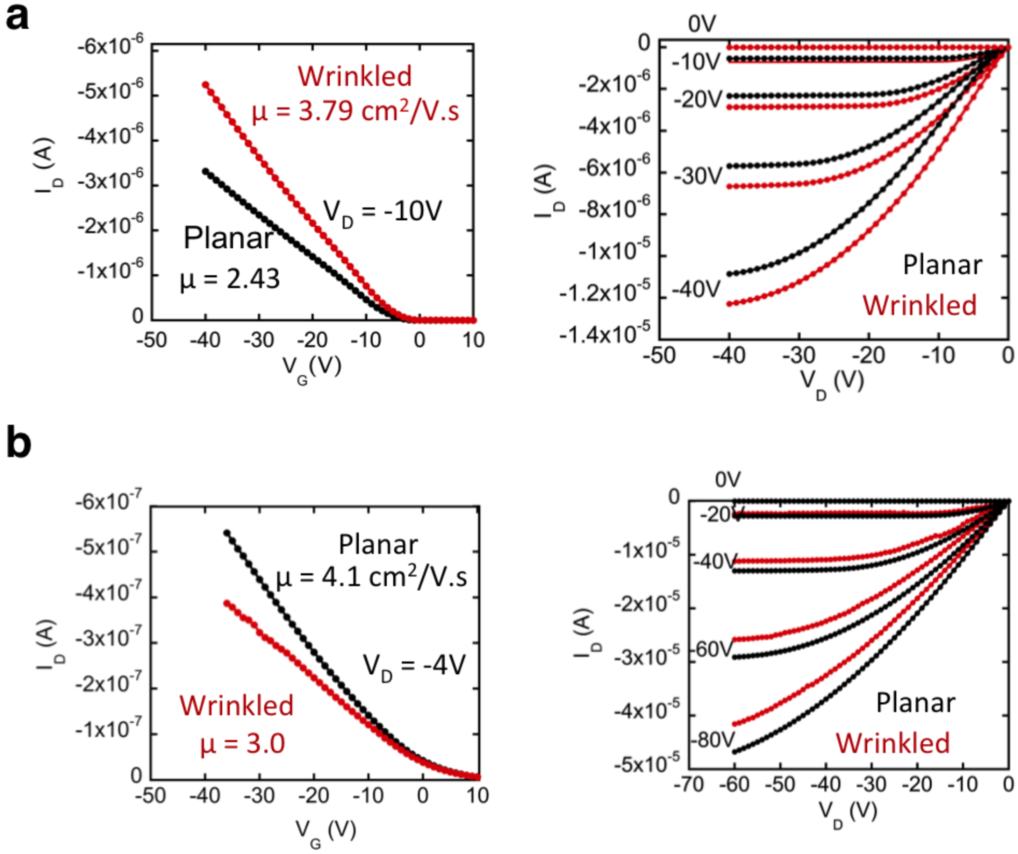

Supplementary Figure 2. Representative transfer and output characteristics of single crystal transistor on elastomeric substrate for a) Mobility increase and b) Mobility decrease upon wrinkling.

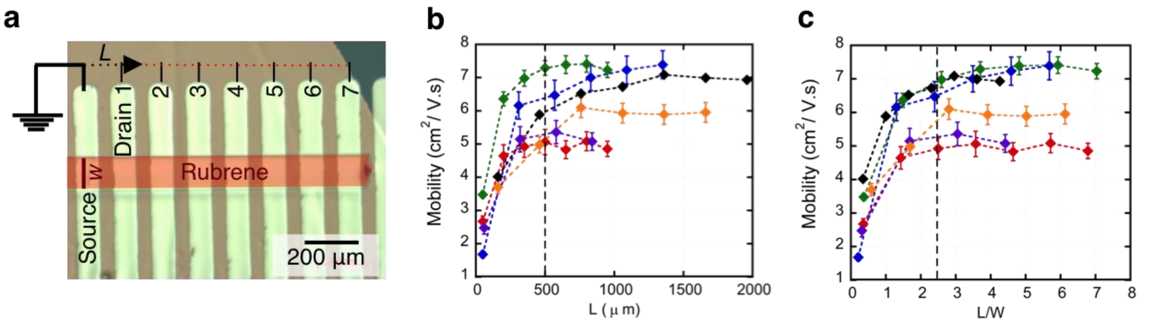

Supplementary Figure 3. Contact-resistance-limited mobility. a) Optical micrograph of representative single crystal FET with multiple top contact source and drain electrodes allowing for multiple channel length testing on same crystal sample. The crystal is artificially highlighted for clarity. b) Mobility trend as a function of channel length for six different rubrene crystals. Different colors represent different samples. Green markers represent device show in (a). Mobility changes are negligible above a channel aspect length of approximately 500 μm. Error bars in mobility capture the variability in six independent measurements from at different drain voltages in the linear regime, -5 V to -15 V at -2 V steps. c) Mobility trend as a function of channel aspect ratio (channel Length/Width) for six different rubrene crystals. Mobility changes are negligible above a channel aspect ratio of approximately 2.5.

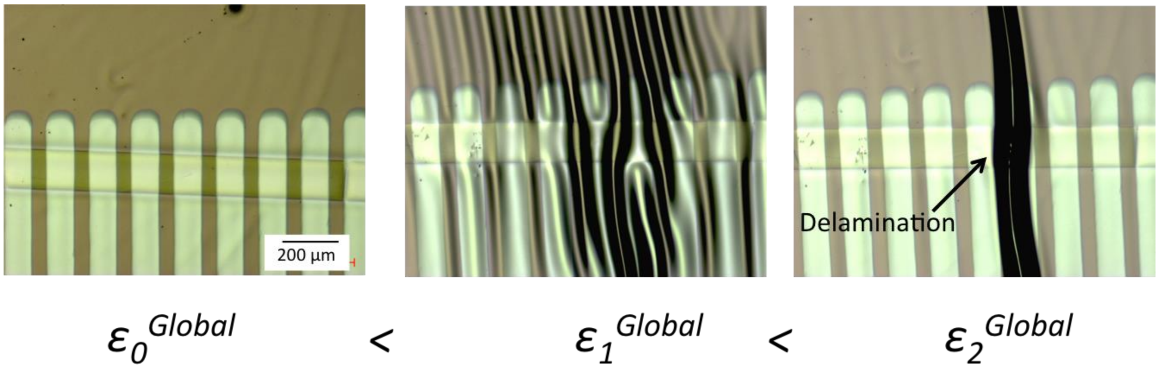

Supplementary Figure 4. Delamination of wrinkled SCFET.

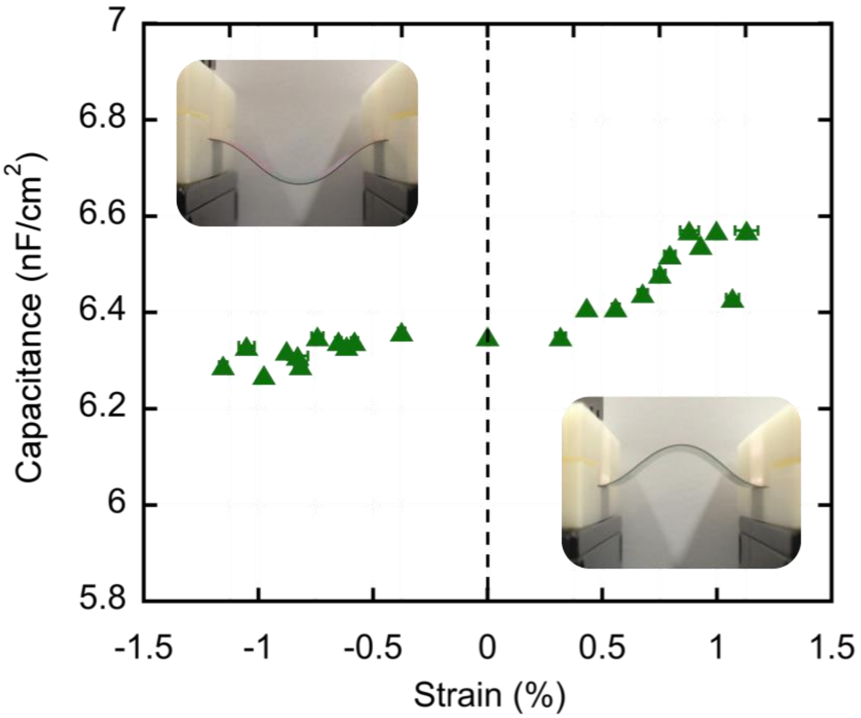

Supplementary Figure 5. Bending parylene capacitor. Parylene capacitance change measured as a function of global bending of parallel plate capacitor. Strain calculated from different bending radii.

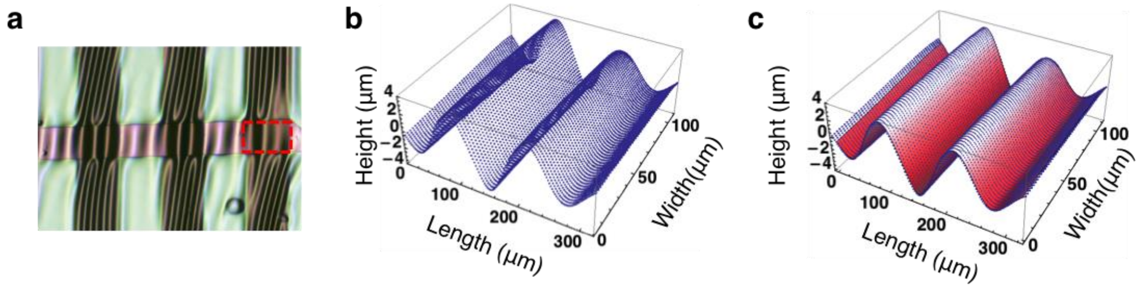

Supplementary Figure 6. Topographic data analysis. a) Optical micrograph of wrinkled single crystal FET. Surface features of wrinkled transistors are measured using optical profilometry. b) 3D plot of x, y, z data obtained from optical profilometry. This data corresponds to dotted area in (a). c) A function of x and y is interpolated through all the surface points. An interpolated function facilitates the manipulation of topographic data using calculus.

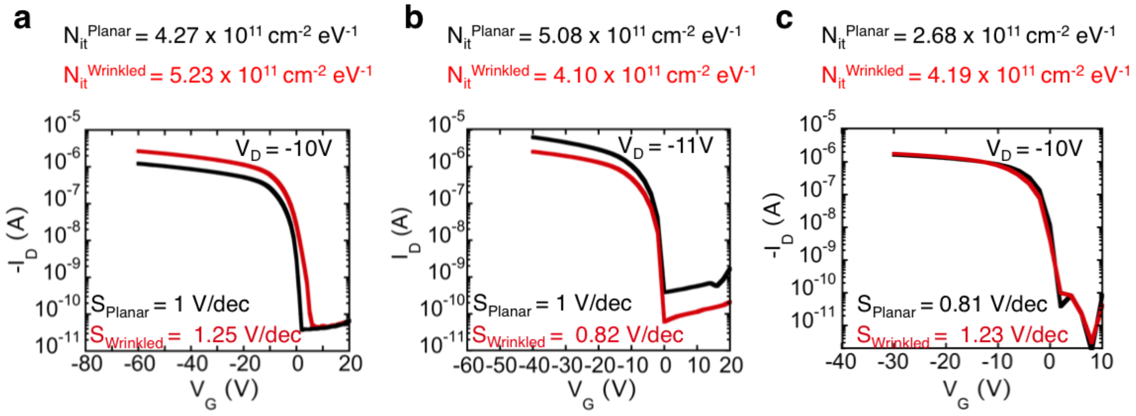

Supplementary Figure 7. Density of interfacial electronic trap states determination.

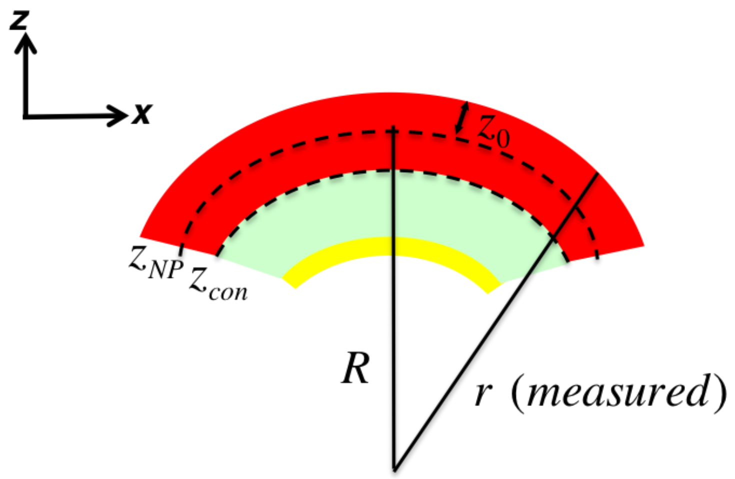

Supplementary Figure 8. Bending of multilayered film.

Supplementary Table 1. Experimental parameters for SCFETs devices tested

$\Delta\mu/\mu_0$	Micrograph	L (μm)	W (μm)	L/W	t_{Au_gate} (nm)	$t_{Parylene}$ (nm)	$t_{Rubrene}$ (nm)	Z_{np}* (nm)
-0.02	Fig 3d 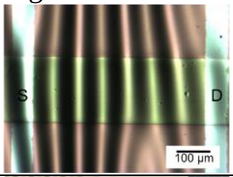	497	190	2.61	40	1074	646	1001
-0.05	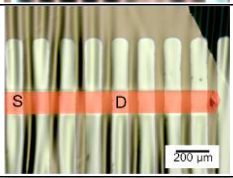	500	135	3.70	40	1125	235	660
-0.05	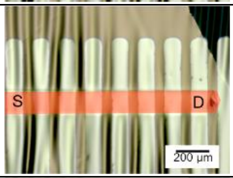	950	135	7.03	40	1125	235	660
-0.06	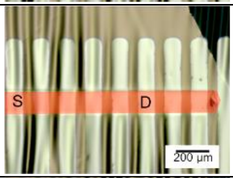	650	135	4.81	40	1125	235	660
-0.09	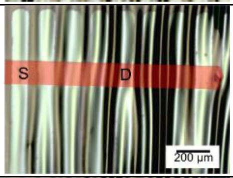	500	135	3.70	40	1125	235	660
-0.11	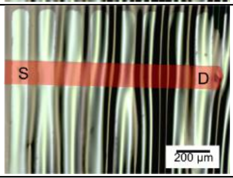	950	135	7.03	40	1125	235	660
-0.37	** 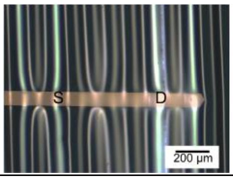	472	69	6.86	40	1055	839	1036
-0.9	Fig 3c** 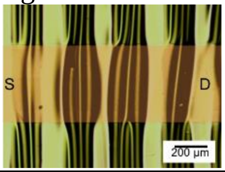	1063	460	2.31	40	520	250	260

**$\Delta\mu/\mu_0$**	**Micrograph**	**L (µm)**	**W (µm)**	**L/W**	**t_{Au_gate} (nm)**	**$t_{Parylene}$ (nm)**	**$t_{Rubrene}$ (nm)**	**Z_{np}* (nm)**
0.025	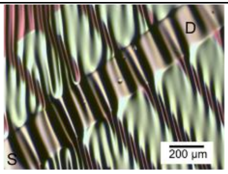	1350	237	5.68	40	703	680	781
0.41	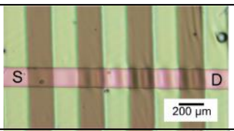	1400	150	9.33	40	685	890	900
0.47	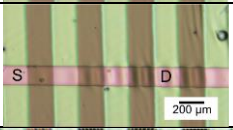	1000	150	6.67	40	685	890	900
0.6	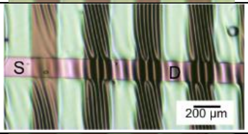	1000	150	6.67	40	685	890	900
0.60	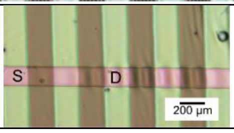	600	150	4	40	685	890	900
0.95	Fig 4d 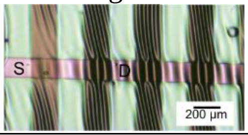	600	150	4	40	685	890	900
1.8	Fig 3b* * 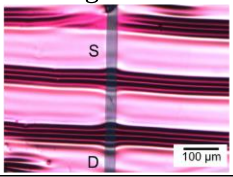	225	38	5.96	35	536	173	322
N/A	Fig 2a** 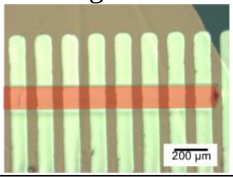	50-950	135	0.37-7.03	40	1125	235	660

*Neutral plane position. Vertical distance from elastomer/transistor interface
**Crystal position has been artificially colorized for clarity.

Supplementary Note 1. Density of interfacial electronic trap states determination

Supplementary Fig. 7 shows the density of interfacial electronic trap states, N_{it}, of the devices shown in Fig. 3. N_{it} is estimated by using:

$$N_{it} = \frac{C_i}{q^2} = \left(\frac{Sq}{k_B T \ln(10)} - 1 \right), \tag{1}$$

where C_i is the capacitance of parylene per unit area, q is the elementary charge, k_B is the Boltzmann constant and T is temperature (300 K).

Small differences in trap state densities are observed between the planar and wrinkled configurations of all devices. No clear correlation between mobility change and N_{it} are observed. Therefore, trap state density does to seem to be the dominant effect in the overall mobility change after wrinkling.

Supplementary Note 2. Bending strain correction

The bending strain of a plate is proportional to the curvature of the neutral plane:

$$e_{xx}^{bending} \mu - zK_{NP} \mu - z\frac{1}{R} \tag{2}$$

where z is the vertical coordinate through the thickness of the plate and K_{NP} corresponds to the curvature of the neutral plane (see Supplementary Fig. 8). The topography of the wrinkled transistors in our study is measured using an optical profilometer, hence, only the out-of-plane deflection of the surface, and therefore, only radius of curvature of the

surface is known. The bending strain expression was corrected to accurately express the bending strain in the conductive channel in terms of the surface curvature.

Let $z_0 = z_{FET} - z_{NP}$, $R = r - z_0$ and $z = z_c - z_{NP}$

$$k = \frac{1}{r} = \frac{\P^2 w(x,y)}{\P x^2} \tag{3}$$

$$K_{NP} = \frac{1}{R} = \frac{1}{r - z_0} = \frac{k}{1 - k z_0} \tag{4}$$

Equation 4 can be written in terms of the second derivative of the out-of-plane displacement:

$$K_{NP} = \left[\frac{\partial^2 w(x,y)}{\partial x^2}\right]\left[1 - \frac{\partial^2 w(x,y)}{\partial x^2} z_0\right]^{-1} \tag{5}$$

The bending strain is then:

$$e_{xx}^{bending}(x,y,z) \propto - z\left[\frac{\partial^2 w(x,y)}{\partial x^2}\right]\left[1 - \frac{\partial^2 w(x,y)}{\partial x^2} z_0\right]^{-1} \tag{6}$$

Writing the contribution of bending strain, together with the von Karman nonlinear elastic plate model gives the full expression for local strain in a wrinkled plate:

$$e_{xx}^{bending}(x,y,z) = e_0 + \frac{1}{2}\left[\frac{\partial w(x,y)}{\partial x}\right]^2 - z\left[\frac{\partial^2 w(x,y)}{\partial x^2}\right]\left[1 - \frac{\partial^2 w(x,y)}{\partial x^2} z_0\right]^{-1} \tag{7}$$
